# Declining Prevalence of *Trichomonas vaginalis* Diagnosed by Wet Mount in a Cohort of U.S. Women With and Without HIV

**DOI:** 10.1089/jwh.2023.0263

**Published:** 2024-03-08

**Authors:** Elizabeth M. Daubert, Jodie Dionne, Jessica Atrio, Andrea K. Knittel, Seble G. Kassaye, Dominika Seidman, Amanda Long, Susan Brockmann, Igho Ofotokun, Margaret A. Fischl, L. Stewart Massad, Kathleen M. Weber

**Affiliations:** ^1^ Cook County Health/Hektoen Institute of Medicine, Chicago, Illinois, USA.; ^2^ Department of Medicine, University of Alabama at Birmingham, Birmingham, Alabama, USA.; ^3^ Montefiore Hospital & Albert Einstein College of Medicine, Bronx, New York, USA.; ^4^ Department of Obstetrics and Gynecology, University of North Carolina at Chapel Hill School of Medicine, Chapel Hill, North Carolina, USA.; ^5^ Department of Medicine, Georgetown University, Washington, District of Columbia, USA.; ^6^ Department of Obstetrics, Gynecology & Reproductive Sciences, University of California San Francisco, San Francisco, California, USA.; ^7^ Department of Epidemiology, Johns Hopkins University Bloomberg School of Public Health, Baltimore, Maryland, USA.; ^8^ State University of New York (SUNY) Health Sciences University, Brooklyn, New York, USA.; ^9^ Division of Infectious Diseases, Department of Medicine, Emory University School of Medicine, Atlanta, Georgia, USA.; ^10^ Division of Infectious Diseases, University of Miami Miller School of Medicine, Miami, Florida, USA.; ^11^ Department of Obstetrics & Gynecology, Washington University School of Medicine, St. Louis, Missouri, USA.

**Keywords:** *Trichomonas vaginalis*, vaginal discharge, human immunodeficiency virus infection in women

## Abstract

**Background:**

Women living with HIV (WLWH) are often coinfected with *Trichomonas vaginalis* (TV), and annual screening is recommended. Our goal was to assess differences in TV prevalence at study entry and over time in enrollment cohorts of the Women's Interagency HIV Study.

**Methods:**

In a multisite study, TV was diagnosed by wet mount microscopy. Prevalence was determined across four enrollment waves: 1994–1995, 2001–2002, 2011–2012, and 2013–2015. Generalized estimating equation multivariable logistic regression models assessed changes in visit prevalence across waves after controlling for HIV disease severity and other risks.

**Results:**

At 63,824 person-visits (3,508 WLWH and 1,262 women without HIV), TV was diagnosed by wet mount at 1979 visits (3.1%). After multivariable adjustment, HIV status was not associated with TV detection, which was more common among younger women, women with multiple partners, and irregular condom use. All enrollment waves showed a decline in TV detection over time, although *p*-value for trend did not reach significance for most recent waves. To explore the potential utility of screening among WLWH, we assessed rates of TV detection among women without appreciable vaginal discharge on examination. Initial TV prevalence among asymptomatic women was 3.5%, and prevalence decreased to 0.5%–1% in the most recent wave (2013–2015) (*p*-trend <0.0001).

**Conclusions:**

In this cohort, TV rates are low among WLWH, and HIV does not increase TV risk. Screening may benefit newly diagnosed WLWH, women with risk factors, or those receiving care sporadically but is unlikely to further reduce the low rate of TV among women in care, especially older women without multiple partners.

The clinical trials registration number for WIHS is NCT00000797.

## Introduction

*T**richomonas vaginalis* (TV) coinfection is identified in a substantial minority of women living with HIV (WLWH), with prevalence rates in the range of 6%–17%.^1–5^ Although rarely a cause of invasive infection, TV can cause a vaginal discharge, dysuria, and genital irritation, and is associated with increased risk of HIV acquisition.^6^ TV can predispose pregnant women to preterm rupture of membranes and preterm labor. Among WLWH, TV has been linked to risk of pelvic inflammatory disease and the transmission of HIV.^6,7^ The Centers for Disease Control and Prevention (CDC) recommends annual screening of WLWH for TV, although the yield of screening among women without symptomatic or clinically evident discharge is unclear.^8^

HIV infection does not appear to impact prevalence or post-treatment persistence of TV.^2,9,10^ Rather, risk factors for TV include younger age, non-White ethnicity, two or more sexual partners, irregular condom use, smoking, and drug or alcohol use; hormonal contraception has been reported to be protective.^2^

The epidemiology and burden of TV among WLWH across time in a cohort of women with HIV have not been recently described. Using data from initial enrollees, Watts et al. reported in 2006 that TV prevalence decreased across time among both women with HIV and uninfected women, but those results only extended through 9 years of follow-up.^2^ This study could not identify an irreducible minimum TV prevalence, suggesting repeated reinfection, inadequate treatment, or an effect of immunosuppression on TV clearance not previously seen. Whether TV prevalence varies across U.S. regions has not been explored.

The main objective of this study was to assess how TV prevalence changed across time during 20 years of follow-up in the Women's Interagency HIV Study (WIHS). We also assessed prevalence across regions and sequential enrollment cohorts to explore geographic and secular trends in TV prevalence. In a subgroup of asymptomatic WLWH without clinically evident discharge, we calculated TV prevalence to evaluate the yield of screening.

## Methods

WIHS is an ongoing U.S. multicenter prospective cohort investigation of HIV and related health conditions, which recruited WLWH and demographically similar women without HIV, now incorporated with the Multicenter AIDS Cohort Study (MACS) into the MACS/WIHS Combined Cohort Study. The protocols, recruitment processes, procedures, and baseline results of the WIHS have been described.^11–13^ Enrollment began before the widespread introduction of combination antiretroviral therapy (cART) with 2,623 women (2,054 with HIV and 569 without) in 1994–1995 at six study consortia (Bronx, Brooklyn, Chicago, Los Angeles, San Francisco, and Washington, DC).

The cohort was expanded by an additional 1,143 women (737 with HIV and 406 without) during 2001–2002. The WIHS was augmented in 2011–2012 by 371 additional women (276 with HIV and 95 without) to account for attrition and most recently by 845 women (611 with HIV and 234 without) from newly funded study sites in the Southern United States (Chapel Hill, Miami, Atlanta, Jackson, and Birmingham) in 2013–2015. As of late 2016, when routine TV assessment with wet mount was discontinued, 1,268 women had died, 130 had withdrawn from the study, 806 had been discontinued for administrative/funding reasons, 415 had been lost to follow-up, and 2,363 were being actively followed.

Local human subjects committees approved study protocols, and written informed consent was obtained from all participants. At entry and each semiannual visit, questionnaires assessed demographic and medical history, and examinations were performed, including pelvic examinations with cervicovaginal specimen collection. Until September 30, 2016, cervicovaginal samples were assessed with screening wet mount microscopy, and TV was diagnosed when motile protozoa were seen. Information about diagnosis and treatment of gynecologic conditions occurring between visits was not reliably collected. HIV serostatus was determined by ELISA with confirmatory testing at study entry for all participants and semiannually thereafter for those initially seronegative.

Although follow-up continues, data from participant visits spanning the interval from October 1, 1994, through September 30, 2016, were included in this analysis. Participant visits were included in the analyses if they had available data on TV and the covariates of interest included in Table 1. Visits at which participants reported use of vaginal medications within 48 hours before the visit were excluded. For Visits 1–8, characteristic discharge was considered present when recorded as any volume greater than none or small. For Visits 9 and following, discharge was considered present when the examining clinician documented that vaginal discharge was increased above normal. We did not consistently collect information about treatment for TV.

**Table 1.** Characteristics of Women Assessed for *Trichomonas vaginalis*^a^ Across Study Visits in the Women's Interagency HIV Study, 1994–2016 (*n* = 63,824)

*N *(%)	TV at visit (*n* = 1,979)	No TV at visit (*n* = 61,845)	*p* ^b^	Unadjusted OR*^c^*(95% CI)	Adjusted OR*^d^* (95% CI)
Recruitment wave					
1994/95	1,194 (60.3)	38,412 (62.1)	**<0.0001**	Reference	Reference
2001/02	509 (25.7)	17,690 (28.6)		**0.80 (0.67–0.96)**	**0.74 (0.62–0.89)**
2011/12	103 (5.2)	2,603 (4.2)		1.07 (0.79–1.46)	1.02 (0.75–1.38)
2013/15	173 (8.8)	3,140 (5.1)		**1.58 (1.27–1.95)**	**1.39 (1.12–1.74)**
HIV serostatus					
Seropositive	1,299 (65.6)	44,747 (72.4)	**<0.0001**	**0.82 (0.70–0.97)**	0.90 (0.76–1.06)
Seronegative	680 (34.4)	17,098 (27.6)		Reference	Reference
Age (years)					
<30	206 (10.4)	5,600 (9.1)	**<0.0001**	**2.59 (2.04–3.29)**	**2.00 (1.51–2.65)**
30–39	712 (36.0)	18,384 (29.7)		**2.57 (2.11–3.12)**	**1.98 (1.59–2.47)**
40–49	773 (39.1)	22,380 (36.2)		**2.06 (1.71–2.47)**	**1.65 (1.35–2.01)**
50+	288 (14.5)	15,481 (25.0)		Reference	Reference
Race/ethnicity					
Non-Hispanic African American	1,494 (75.5)	31,978 (51.7)	**<0.0001**	**4.93 (3.43–7.11)**	**4.40 (3.09–6.25)**
Hispanic	196 (9.9)	15,781 (25.5)		1.30 (0.86–1.96)	1.29 (0.86–1.93)
Non-Hispanic Other	230 (11.6)	7,181 (11.6)		**3.34 (2.21–5.06)**	**3.35 (2.26–4.98)**
Non-Hispanic White	59 (3.0)	6,905 (11.2)		Reference	Reference
Household income, ≤$18,000 annual	1,618 (81.8)	40,689 (65.8)	**<0.0001**	**1.67 (1.48–1.89)**	**1.46 (1.28–1.67)**
Education level, <HS	869 (43.9)	22,635 (36.6)	**<0.0001**	**1.35 (1.16–1.56)**	**1.16 (1.00–1.34)**
Drinks per week, >7	417 (21.1)	6,503 (10.5)	**<0.0001**	**1.76 (1.52–2.04)**	**1.29 (1.11–1.50)**
Current smoker	1,434 (72.5)	27,739 (44.9)	**<0.0001**	**2.49 (2.20–2.83)**	**1.88 (1.64–2.15)**
Depressive symptoms (CES-D), ≥16	1,035 (52.3)	22,350 (36.1)	**<0.0001**	**1.65 (1.49–1.81)**	**1.40 (1.26–1.56)**
Sexual activity/condom use					
No recent vaginal sex	427 (21.6)	22,413 (36.2)	**<0.0001**	Reference	Reference
Condom use always, ≥1 partner	691 (34.9)	19,902 (32.2)		**1.69 (1.48–1.93)**	**1.39 (1.21–1.61)**
Sometimes/never condom use, 1 partner	562 (28.4)	15,802 (25.6)		**1.68 (1.44–1.96)**	**1.41 (1.19–1.67)**
Sometimes/never condom use, >1 partner	299 (15.1)	3,728 (6.0)		**3.11 (2.59–3.72)**	**1.80 (1.48–2.19)**
Had sex for drugs, money, and/or shelter, yes	224 (11.3)	1,723 (2.8)	**<0.0001**	**3.26 (2.69–3.95)**	**1.89 (1.56–2.30)**
Hormonal contraceptive use, yes	88 (4.5)	4,482 (7.3)	**<0.0001**	**0.77 (0.64–0.92)**	**0.68 (0.55–0.83)**
Menopausal status, menopausal	398 (20.1)	17,985 (29.1)	**<0.0001**	**0.55 (0.47–0.63)**	**0.77 (0.65–0.91)**
Enrollment site/location^e^					
Bronx, NY	300 (15.2)	12,593 (20.3)	**<0.0001**	Reference	
Brooklyn, NY	304 (15.3)	11,774 (19.0)		1.07 (0.82–1.39)	
Washington DC	275 (13.9)	8,475 (13.7)		**1.44 (1.10–1.88)**	
Los Angeles, CA	135 (6.8)	9,138 (14.8)		**0.63 (0.46–0.86)**	
San Francisco, CA	398 (20.1)	8,464 (13.7)		**1.95 (1.49–2.55)**	
Chicago, IL	394 (19.9)	8,261 (13.4)		**2.02 (1.56–2.63)**	
Chapel Hill, NC	19 (1.0)	743 (1.2)		1.03 (0.62–1.70)	
Atlanta, GA	93 (4.7)	998 (1.6)		**3.63 (2.62–5.03)**	
Miami, FL	22 (1.1)	545 (0.9)		1.62 (0.92–2.86)	
Birmingham, AB	13 (0.7)	407 (0.7)		1.31 (0.74–2.32)	
Jackson, MS	26 (1.3)	447 (0.7)		**2.33 (1.29–4.22)**	

Bold highlights significant comparison.

^a^ Determined through wet prep/saline mount.

^b^ *p*-Values obtained from chi-square tests.

^c^ Results are from the GEE model to control for repeat measures.

^d^ Results are from the multivariable GEE model. All variables listed were included in the model with the exception of enrollment site/location.

^e^ Not included in multivariable model due to collinearity.

GEE, generalized estimating equation; TV, *Trichomonas vaginalis*.

Covariates of interest included enrollment wave; HIV serostatus; age at visit; self-reported race/ethnicity; household income; educational attainment; alcoholic drinks per week; smoking status; depressive symptoms (CES-D); reported sexual activity and condom use (no recent vaginal sex, always condom use and one or more partners, never/sometimes condom use and one partner, never/sometimes condom use and more than one partner); reported sex for money, drugs, and/or shelter; hormonal contraceptive use, self-reported menopausal status; and enrollment WIHS site or subsite.

Co-occurrent bacterial vaginosis (BV) was determined if vaginal pH >4.5 and two of three other Amsel criteria were met: clue cells on wet mount, positive amine odor after KOH application to discharge, and characteristic discharge. Among analyses limited to WLWH, additional variables included CD4 count (cells/mm^3^), HIV viral load detection at visit, and cART use at visit.

Bivariate analyses using chi-square tests assessed the relationship between TV at a visit and sociodemographic and clinical covariates. To account for repeat measures within participants, generalized estimating equations (GEEs) adjusted odds ratios (ORs), and 95% confidence intervals (CIs) were calculated to determine predictors of TV. In multivariable (GEE) logistic regression models, overall and among women with HIV only, all variables associated with TV in the univariate GEE-adjusted models were included. Enrollment site was excluded from multivariable analyses due to collinearity with wave—since all participants in the 2013–2015 enrollment were from Southern sites, and all Southern site participants included only women in that wave.

In addition, regional differences in TV prevalence were calculated in univariate analyses. The prevalence of TV in women at each visit, stratified by WIHS enrollment wave, and the proportion of TV among women with HIV without characteristic discharge were also assessed. Finally, TV prevalence by age and subanalysis of women of reproductive age (≤40 years) were examined. All analyses were performed using SAS software, version 9.4 (SAS Institute, Inc., Cary, NC). Statistical significance was set at *p* < 0.05.

## Results

During follow-up, there were 78,617 person-visits that included wet mount interpretation. Of these, 13,480 visits (17%) were excluded for missing data, and 1,313 visits (2%) were excluded for using vaginal medications within 48 hours of a visit (3%; 172 women were excluded entirely). This left 63,824 person-visits (4,770 women: 3,508 WLWH and 1,262 women without HIV), which included 1,979 (3.1%) TV-positive and 61,845 (96.9%) TV-negative visits. Co-occurrent BV was seen in 1,001 (50.6%) of the positive TV visits.

Table 1 shows how TV was detected across women with various demographic, behavioral, and medical characteristics. After multivariable adjustment, TV detection was more common among women <50 years of age, non-Hispanic African American and non-Hispanic other women, women with annual incomes <$18,000, women who acknowledged drinking and smoking, and clinically significant depressive symptoms. Women who reported multiple partners and irregular condom use had the highest probability of TV detection, while those who reported no vaginal sex had the lowest risk; transactional sex was an independent correlate of TV detection. Even after controlling for age and other covariates, women using hormonal contraception and those reporting menopause had lower TV detection risk than others.

HIV status was not associated with TV detection in multivariable analysis. However, since screening is recommended for WLWH, we assessed correlates of TV detection among these women. As shown in Table 2, TV detection in WLWH was associated with risk factors similar to those in the larger dataset: younger age, non-Hispanic African American and non-Hispanic Other race/ethnicity, lower income, less than a high school education, harmful drinking and smoking, and clinically significant depressive symptoms.

**Table 2.** Characteristics of HIV-Positive Women Assessed for *Trichomonas vaginalis*^a^ Across Study Visits in the Women's Interagency HIV Study (*n* = 46,046)

*N *(%)	TV at visit (*n* = 1,299)	No TV at visit (*n* = 44,747)	*p* ^b^	Unadjusted OR*^c^* (95% CI)	Adjusted OR*^d^* (95% CI)
Recruitment wave					
1994/95	809 (62.3)	29,245 (65.4)	**<0.0001**	Reference	Reference
2001/02	306 (23.6)	11,318 (25.3)		0.86 (0.69–1.07)	0.99 (0.80–1.22)
2011/12	63 (4.8)	1,950 (4.3)		0.96 (0.67–1.38)	1.34 (0.93–1.92)
2013/15	121 (9.3)	2,234 (5.0)		**1.70 (1.31–2.21)**	**2.40 (1.83–3.14)**
Age (years)					
<30	133 (10.2)	3,002 (6.7)	**<0.0001**	**3.37 (2.56–4.44)**	**1.94 (1.37–2.75)**
30–39	518 (39.9)	13,208 (29.5)		**2.95 (2.38–3.65)**	**1.84 (1.40–2.41)**
40–49	490 (37.7)	16,929 (37.8)		**2.07 (1.69–2.53)**	**1.48 (1.15–1.91)**
50+	158 (12.2)	11,608 (26.0)		Reference	Reference
Race/ethnicity					
Non-Hispanic African American	982 (75.6)	23,094 (51.6)	**<0.0001**	**4.94 (3.22–7.59)**	**3.97 (2.64–5.98)**
Hispanic	120 (9.2)	11,503 (25.7)		1.23 (0.75–2.01)	1.11 (0.69–1.80)
Non-Hispanic Other	153 (11.8)	4,815 (10.8)		**3.71 (2.28–6.05)**	**3.80 (2.40–6.02)**
Non-Hispanic White	44 (3.4)	5,335 (11.9)		Reference	Reference
Household income, ≤$18,000 annual	1,068 (82.2)	30,003 (67.1)	**<0.0001**	**1.77 (1.52–2.07)**	**1.46 (1.22–1.74)**
Education level, <HS	586 (45.1)	16,756 (37.5)	**<0.0001**	**1.37 (1.15–1.63)**	**1.21 (1.03–1.44)**
Drinks per week, >7	250 (19.3)	3,854 (8.6)	**<0.0001**	**1.97 (1.64–2.37)**	**1.30 (1.08–1.58)**
Current smoker	895 (68.9)	19,011 (42.5)	**<0.0001**	**2.47 (2.12–2.87)**	**1.71 (1.44–2.01)**
Depressive symptoms (CES-D), ≥16	694 (53.4)	16,834 (37.6)	**<0.0001**	**1.65 (1.47–1.86)**	**1.31 (1.15–1.50)**
Sexual activity/condom use					
No recent vaginal sex	335 (25.8)	17,204 (38.5)	**<0.0001**	Reference	Reference
Condom use always, ≥1 partner	544 (41.9)	17,064 (38.1)		**1.56 (1.34–1.81)**	**1.25 (1.06–1.48)**
Sometimes/never condom use, 1 partner	286 (22.0)	8,892 (19.9)		**1.59 (1.32–1.91)**	**1.30 (1.06–1.60)**
Sometimes/never condom use, >1 partner	134 (10.3)	1,587 (3.5)		**3.37 (2.66–4.27)**	**1.71 (1.32–2.20)**
Had sex for drugs, money, and/or shelter, yes	146 (11.2)	1,062 (2.4)	**<0.0001**	**3.93 (3.12–4.95)**	**2.07 (1.64–2.62)**
Hormonal contraceptive use, yes	59 (4.5)	2,923 (6.5)	**0.004**	0.80 (0.63–1.01)	**0.71 (0.54–0.93)**
Menopausal status, menopausal	232 (17.9)	13,831 (30.9)	**<0.0001**	**0.50 (0.42–0.58)**	**0.72 (0.59–0.90)**
Enrollment site/location^e^					
Bronx, NY	184 (14.2)	8,568 (19.2)	**<0.0001**	Reference	
Brooklyn, NY	201 (15.5)	8,555 (19.1)		1.06 (0.78–1.43)	
Washington DC	196 (15.1)	6,162 (13.8)		**1.50 (1.09–2.06)**	
Los Angeles, CA	70 (5.4)	6,894 (15.4)		**0.48 (0.32–0.72)**	
San Francisco, CA	248 (19.1)	5,968 (13.3)		**1.94 (1.43–2.64)**	
Chicago, IL	279 (21.5)	6,366 (14.2)		**1.98 (1.45–2.69)**	
Chapel Hill, NC	15 (1.1)	550 (1.2)		1.16 (0.65–2.07)	
Atlanta, GA	60 (4.6)	673 (1.5)		**3.67 (2.43–5.56)**	
Miami, FL	16 (1.2)	373 (0.8)		1.84 (0.92–3.68)	
Birmingham, AB	10 (0.8)	300 (0.7)		1.46 (0.75–2.83)	
Jackson, MS	20 (1.5)	338 (0.8)		**2.54 (1.26–5.10)**	
CD4 count (cells/mm^3^)					
<200	331 (25.5)	6,725 (15.0)	**<0.0001**	**1.95 (1.62–2.35)**	**1.64 (1.34–2.00)**
200–499	549 (42.3)	17,643 (39.4)		**1.41 (1.23–1.62)**	**1.20 (1.03–1.40)**
≥500	419 (32.2)	20,379 (45.6)		Reference	Reference
Detectable viral load at visit, yes	1,000 (77.0)	24,408 (54.6)	**<0.0001**	**2.13 (1.88–2.42)**	**1.35 (1.14–1.59)**
HAART use at visit, yes	511 (39.3)	27,853 (62.3)	**<0.0001**	**0.46 (0.41–0.52)**	**0.71 (0.60–0.83)**

Bold highlights significant comparison.

^a^ Determined through wet prep/saline mount.

^b^ *p*-Values obtained from chi-square tests.

^c^ Results are from the GEE model to control for repeat measures.

^d^ Results are from the multivariable GEE model. All variables listed were included in the model with the exception of enrollment site/location.

^e^ Not included in multivariable model due to collinearity.

HAART, highly active antiretroviral therapy.

Again, single partner and consistent condom use were associated with TV risk intermediate between abstinence and irregular condom use with multiple partners; transactional sex was also linked to TV detection. Hormonal contraceptive use and reported menopause were linked to lower TV detection risk. Indicators of more severe HIV disease, including lower CD4 count and detectable HIV RNA, were linked to higher risk of TV detection, while use of highly active antiretroviral therapy (HAART) was protective independent of these factors.

**FIG. 1.**
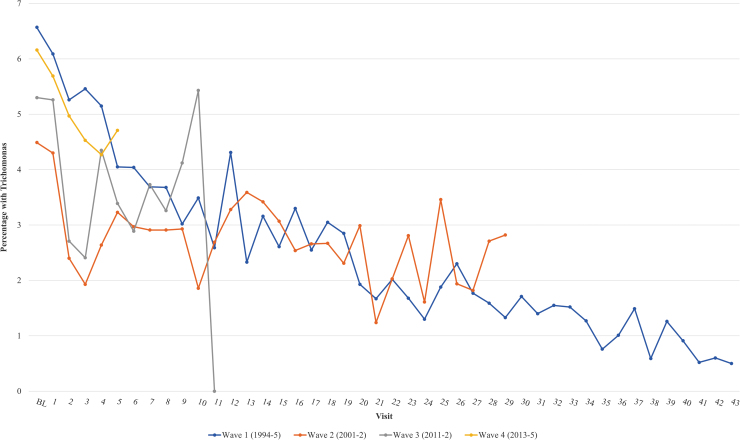
Proportion of women diagnosed with *Trichomonas vaginalis* across all four enrollment waves of the Women's Interagency HIV Study (1994–5, 2001–2, 2011–2, 2013–5). Visit number reflects the number of visits attended by individuals in each enrollment wave; missed visits are not included (*p*-trend: Wave 1 < 0.0001, Wave 2 0.009, Wave 3 0.363, Wave 4 0.100).

Figure 1 shows how TV detection changed across time in sequential semiannual visits in the four enrollment waves of the WIHS. In Wave 1, TV detection decreased across study visits from an initial high of ∼7% to <1%. Although follow-up has been shorter for subsequent waves, each showed a similar decline, although *p*-value for trend did not reach significance for Wave 3 or Wave 4. At initial enrollment assessment, the prevalence of TV was lower in Wave 2 than in other enrollment waves. Recalculating curves after adding back women visits previously excluded for missing data did not result in meaningful changes.

We explored whether women from different U.S. regions differed in TV prevalence. In an analysis adjusted for repeated measures but not other potential covariates, TV prevalence varied among sites. TV prevalence was higher among women from the U.S. Midwest (Chicago; OR 1.76, 95% CI 1.43–2.18) and South (Chapel Hill, Atlanta, Miami, Birmingham, Jackson combined; OR 1.90, 95% CI 1.52–2.36) compared with a reference group from Eastern sites (Brooklyn, Bronx, District of Columbia). When sites were combined, TV risks among women from West Coast sites were similar to those among Eastern women (OR 1.09, 95% CI 0.91–1.32), but prevalence was higher in San Francisco and lower in Los Angeles.

**FIG. 2.**
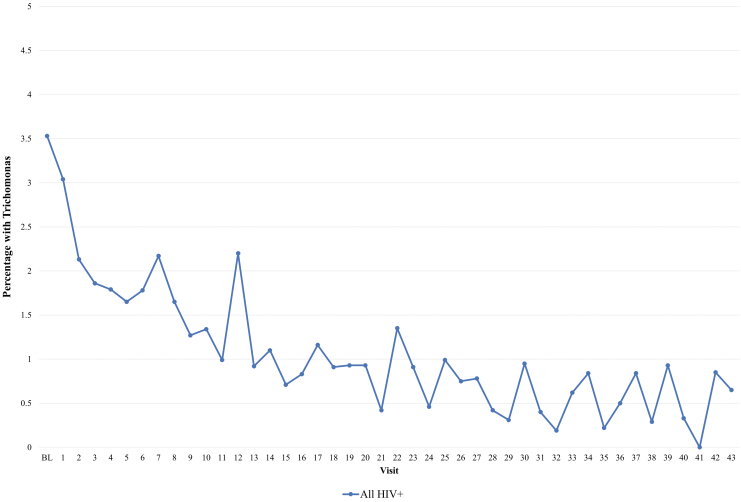
Proportion of women positive for *Trichomonas vaginalis* by wet mount among HIV-positive women without clinical discharge. Visit number reflects the number of visits attended by individuals (*p*-trend <0.0001).

To further explore the potential utility of screening among WLWH, we assessed rates of TV detection among women who did not have appreciable vaginal discharge on examination. Figure 2 shows how TV prevalence declined across time at sequential semiannual visits among all WLWH with clinical findings of no or small discharge volume. Although initial prevalence was 3.5%, with time prevalence decreased to 0.5%–1% (*p*-trend <0.0001). Again, recalculating curves after adding back women visits previously excluded for missing data did not result in meaningful changes.

Finally, we explored the prevalence of TV by age. The prevalence of TV for ages 20–45 years was consistent, between 3.5% and 6.5%, and then declined to 1%–3% at ≥50 years. We also stratified the overall TV prevalence across semiannual visits by age (≤40, >40 years). TV prevalence did not differ by age group, each showed a similar decline over time; initial prevalence was 5.5%–6.5% for both age subgroups and decreased to <1% by the end of follow-up. In addition, in a multivariable-adjusted subanalysis of women of reproductive age (≤40 years) the findings did not result in appreciable differences in the results for the overall analytic sample; TV detection was associated with similar risk factors (data not shown).

## Discussion

In this large U.S. study of WLWH and comparison women without HIV, the prevalence of TV was lower than that previously reported and decreased with time in the study. Several authors using wet mount microscopy found only marginally higher TV prevalence, with rates of 6%–17%^2–5^; of note, Muzny et al. found that prevalence was only 3.5% among asymptomatic WLWH, 20% lower than among women seeking gynecologic care for symptoms.^5^

A decline in TV risk over time was reported by Cu-Uvin and colleagues from the HIV Epidemiology Research Trial, another prospective cohort of WLWH and women without HIV.^3^ Our results expand on and extend the findings of Watts et al. from the initial enrollment cohort, including the decline in TV risk with time in the study.

TV was not associated with HIV status in this cohort, but instead was associated with demographic and behavioral factors linked to HIV, including younger age, non-Hispanic African American race/ethnicity, lower income, and multiple sexual partners in the context of inconsistent condom use. These findings are consistent with prior work,^2,9,10^ and may drive regional differences in TV risk that we observed. Observed race differences are likely multifactorial, and driven by longstanding social and structural inequities.

Multilevel interventions will be necessary to eliminate racism and the associated structural barriers to ensure optimal health for all Black women. We found higher prevalence rates in the South and Midwest compared with the East and West. This may reflect demographic and behavioral differences that differ by region, differences in recruitment strategy or TV treatment, differences in access to safer sex education and services, differential access to expanded Medicaid and health services, and differences in substance use and transactional sex; further sampling of a wider range of sites is needed to explore this finding.

Additional research is needed to determine why TV risk declined across time in this study. The limitations of episodic contact with study participants meant that we were unable to distinguish the relative contributions of treatment, mortality bias, age, enrollment period, and behavior change, especially safer sex practices and abstinence resulting from study participation and study-related and other educational efforts. WIHS research has shown that WLWH are less likely than women without HIV to engage in sexual activity, especially condomless anal or vaginal intercourse,^14^ but all of these factors are likely to have had some impact on declining TV risk in this study.

Several factors may limit the generalizability of our findings. Wet mount assessment is less sensitive than nucleic acid amplification testing (NAAT) for the detection of TV, and our results likely underestimate the true prevalence of TV among women with and without HIV. NAAT is considered the current optimal test for vaginitis testing, given high accuracy and the ability for combination tests for Candida and BV as well as trichomonas. Compared with culture, the sensitivity of wet mount for TV diagnosis has been estimated at 69%; sensitivity for diagnosing coinfections may be lower.^15^

Nevertheless, the International Society for the Study of Vulvovaginal Disease considers wet mount microscopy an accepted diagnostic tool for TV because it is immediately available, inexpensive, allows for simultaneous pH assessment, and often allows the diagnosis of multiple coinfections and disorders.^16^ Newer rapid and point-of-care tests provide immediate results with high sensitivity, and may be useful in settings where NAAT is not available or where quick turnaround is needed because adherence to follow-up recommendations is a concern.^17^ NAAT had not been developed when WIHS was launched in 1994, and this longitudinal cohort offers a window into the temporal dynamics of TV prevalence in at-risk women going back almost three decades.

In addition, in a post-HAART era, this is a cohort of predominantly midlife women aging with HIV, which may limit generalizability to younger WLWH. However, our results from the subanalysis of women of reproductive age produced similar results to the overall analysis. This suggests that age may not be the primary driver of the decline in TV prevalence, but time spent in the study and/or detection and treatment.

Even if true TV prevalence is twice what we estimate in this cohort, routinely screening women without clinical discharge for TV may not yield health benefits for WLWH. This study and others have demonstrated that WLWH do not have elevated TV risk compared with women without HIV; yet current national policy recommends screening for TV among WLWH because they may be at higher risk of complications.^8^ Lazenby et al. found that annual TV screening of WLWH would be cost effective, but our results raise concerns about assumptions used in their modeling study.^18^ These included a 23% rate of TV infection, multiple partners for the majority of women, and high rates of reinfection and persistence.

Prevalence estimates were also drawn from studies that included symptomatic women. Women with low prevalence infection may still benefit from TV screening if harms are substantial and benefits clear. The CDC recommendation for screening WLWH for TV cited a study demonstrating increased vaginal shedding of HIV among women coinfected with TV that decreased after TV therapy, but that study did not assess the utility of annual screening. The CDC guidance also cited studies showing an increased risk of prematurity, preterm rupture of membranes, and low birth weight, but those studies did not recruit WLWH and were completed more than three decades ago, before heterosexual transmission of HIV was common and before antiretroviral therapy had been developed. TV infection may predispose to HIV transmission,^1^ and TV screening may reduce transmission.

Our results suggest that current policy should be critically reappraised. TV screening is indicated for WLWH initiating care, since we found 3.5% had TV by wet mount microscopy and yield is likely to be greater with more advanced molecular testing. Because of the potential perinatal consequences of TV infection, screening pregnant WLWH may be useful. While women with symptoms of vaginitis or clinically evident discharge should be evaluated regardless of HIV status with NAAT assays that include TV assessment,^8^ screening WLWH without discharge visible on examination appears to be of limited utility and should not be recommended for all WLWH. Screening also might have substantial yield for WLWH with sporadic medical encounters, although this should be explored in future studies.

Finally, women with multiple risk factors may benefit from screening, and clinicians in high prevalence settings may incorporate screening regardless of women's HIV status, although research is needed to estimate yield and impact. Investigators might assess the role of TV screening among WLWH with low CD4 counts and detectable HIV RNA levels and targeted screening after behavioral risk assessment. On the contrary, our findings indicate that screening for TV may not offer sufficient benefit for women without risk factors in established long-term clinical relationships.
